# Supplementary material for: Depressive Symptoms in Middle-Aged and Elderly Women Are Associated with a Low Intake of Vitamin B6: A Cross-Sectional Study
Source: Nutrients. 2020 Nov 9;12(11):3437. doi: 10.3390/nu12113437 (PMC7697531; doi:10.3390/nu12113437)
Supplement: Supplementary file 1 [file nutrients-12-03437-s001.pdf]

Table S1. The major 43 nutrients assessed by the brief-type self-administered diet history questionnaire.

Protein	Protein
	Animal protein
	Vegetable protein
Carbohydrate	Carbohydrate
	Soluble dietary fiber
	Insoluble dietary fiber
	Dietary fiber
Fat	Fat
	Animal fat
	Vegetable fat
	Saturated fatty acid
	Polyunsaturated fatty acid
	Cholesterol
	n-3 fatty acid
	n-6 fatty acid
Vitamin	Retinol
	β-Carotene
	Retinol equivalent
	Vitamin D
	α-Tocopherol
	Vitamin K
	Vitamin B_1
	Vitamin B_2
	Niacin
	Vitamin B_6
	Vitamin B_{12}
	Folic acid
	Pantothenic acid
	Vitamin C
Ash	Ash
Mineral	Sodium
	Potassium
	Calcium
	Magnesium
	Phosphorus
	Iron
	Zinc
	Copper
	Manganese
Others	Daidzein
	Genistein
	Alcohol

Table S2. The Menopausal Health-Related Quality of Life Questionnaire

	Physical and psychological symptoms	Score			
		None	Mild	Moderate	Severe
1	I feel nauseous.	3	2	1	0
2	I feel dizzy.	3	2	1	0
3	My limbs are numb.	3	2	1	0
4	I have backache, stiff neck, and joint pain.	3	2	1	0
5	I get tired easily.	3	2	1	0
6	I have headache.	3	2	1	0
7	I go to the restroom a lot.	3	2	1	0
8	I find hot flushes embarrassing.	3	2	1	0
9	I often sweat in my sleep.	3	2	1	0
10	I feel less enthusiastic about things than before.	3	2	1	0
11	Things I used to enjoy have become a bit of a chore.	3	2	1	0
12	I do less than I would like.	3	2	1	0
13	My mood is generally depressed.	3	2	1	0
14	I forget things easily.	3	2	1	0
15	I cannot concentrate easily.	3	2	1	0
16	I upset about small things.	3	2	1	0
17	I feel nervous about small things.	3	2	1	0
18	I am not content with my sexual life.	3	2	1	0
19	I have trouble falling asleep.	3	2	1	0
20	The quality of my sleep is low.	3	2	1	0
21	I am not content with my appearance.	3	2	1	0
	**Life satisfaction**	Definitely false	Probably false	Probably true	Definitely true
1	I am content with my work/housework.	0	1	2	3
2	I find I have the energy to do the things I want	0	1	2	3
3	The future seems hopeful for me.	0	1	2	3
4	I consider my life interesting and stimulating.	0	1	2	3
5	I consider my life worth living.	0	1	2	3
	**Social involvement**	Definitely false	Probably false	Probably true	Definitely true
1	I think it is important to get a lot of recognition in my community, such as Parent-Teacher Organization, reunion, culture center, and women's committee.	0	1	2	3
2	I believe relationships are important in society.	0	1	2	3
		True		False	
3	I believe I make the most of what I've learned in a daily life.	1		0	
4	I believe I do something for people and society.	1		0	

5	I attend meetings or non-religious organizations, such as Parent-Teacher Organization, reunion, and culture center.	1	0
6	I am responsible for the organization.	1	0
7	I have done some volunteer work within a year.	1	0
8	I have been involved in social activities within a year, such as political, civil, and consumer activity.	1	0
9	I have participated in gathering with relatives, peers, and neighbors, within a year.	1	0
10	I have engaged in environmental preservation activities within a year.	1	0
11	I have a relative and peer who help each other.	1	0
12	If I were sick, I could easily find someone to help me.	1	0

Table S3. The differences in daily intake of nutrients between the severity groups

Nutrients	All Participants (N = 289)	Anxiety				Depression			
		Mild (n = 156)	Moderate (n = 83)	Severe (n = 50)	p-Value[a]	Mild (n = 178) (n = 166)	Moderate (n = 74)	Severe (n = 37) (n = 79)	p-Value[a]
Total energy, MJ	6.99 (1.98)	6.83 (1.95)	7.43 (2.07)	6.76 (1.85)	0.083	6.85 (1.90)	7.42 (2.11)	6.78 (2.03)	0.167
Protein, %E	16.3 (3.2)	16.6 (3.4)	15.9 (2.8)	16.2 (2.9)	0.236	16.8 (3.4)	15.5 (2.3)	15.9 (3.3)	0.048
Animal protein, %E	9.4 (3.3)	9.7 (3.3)	9.0 (3.2)	9.1 (3.3)	0.265	9.8 (3.5)	8.6 (2.4)	8.8 (3.5)	0.051
Vegetable protein, %E	6.9 (1.3)	6.9 (1.2)	6.9 (1.2)	7.1 (1.4)	0.845	6.9 (1.3)	6.9 (1.1)	7.0 (1.3)	0.819
Carbohydrate, %E	52.7 (7.9)	52.3 (7.8)	53.2 (8.5)	52.9 (7.0)	0.724	51.7 (7.9)	54.7 (6.9)	53.0 (9.0)	0.027
Soluble dietary fiber, g/MJ	0.50 (0.17)	0.51 (0.18)	0.46 (0.13)	0.51 (0.19)	0.155	0.52 (0.17)	0.46 (0.15)	0.45 (0.15)	0.014
Insoluble dietary fiber, g/MJ	1.35 (0.43)	1.40 (0.46)	1.25 (0.33)	1.36 (0.47)	0.082	1.41 (0.45)	1.25 (0.38)	1.24 (0.36)	0.007
Dietary fiber, g/MJ	1.90 (0.62)	1.97 (0.66)	1.77 (0.49)	1.93 (0.68)	0.102	2.00 (0.65)	1.77 (0.57)	1.74 (0.53)	0.006
Fat, %E	28.0 (5.5)	28.0 (5.5)	27.9 (5.5)	28.2 (5.4)	0.947	28.3 (5.4)	27.5 (5.6)	27.8 (6.0)	0.776
Animal fat, %E	13.0 (4.2)	13.1 (4.1)	12.7 (4.2)	12.9 (4.6)	0.812	13.3 (4.3)	12.3 (3.9)	12.5 (4.5)	0.204
Vegetable fat, %E	15.0 (3.8)	14.9 (4.0)	15.2 (3.4)	15.3 (3.7)	0.593	14.9 (3.8)	15.2 (3.8)	15.2 (3.6)	0.542
Saturated fatty acid, %E	7.7 (1.9)	7.6 (1.9)	7.8 (1.9)	7.6 (1.8)	0.569	7.7 (1.9)	7.7 (1.8)	7.4 (1.8)	0.613
Monounsaturated fatty acid, %E	9.9 (2.2)	9.8 (2.2)	9.9 (2.3)	9.8 (2.3)	0.902	9.9 (2.2)	9.7(2.3)	9.7 (2.4)	0.752
Polyunsaturated fatty acid, %E	6.8 (1.5)	6.8 (1.5)	6.6 (1.3)	7.0 (1.5)	0.473	6.8 (1.4)	6.5 (1.5)	7.0 (1.6)	0.438
Cholesterol, mg/MJ	45.1 (15.5)	45.5 (15.7)	44.5 (15.2)	44.7 (15.7)	0.959	45.7 (15.4)	43.5 (14.2)	44.9 (18.4)	0.429
n-3 fatty acid, %E	1.4 (0.5)	1.5 (0.5)	1.3 (0.4)	1.4 (0.5)	0.061	1.5 (0.5)	1.3 (0.4)	1.4 (0.5)	0.148
n-6 fatty acid, %E	5.3 (1.2)	5.3 (1.2)	5.3 (1.0)	5.6 (1.2)	0.517	5.4 (1.1)	5.2 (1.2)	5.6 (1.3)	0.598
Ash, g/MJ	2.65 (0.53)	2.73 (0.58)	2.50 (0.40)	2.64 (0.50)	0.015	2.7 (0.6)	2.5 (0.4)	2.5 (0.6)	0.005
Sodium, mg/MJ	578.1 (127.8)	592.8 (140.9)	553.0 (102.4)	574.1 (117.6)	0.117	587.4 (139.4)	561.6 (89.4)	566.7 (133.1)	0.582
Potassium, mg/MJ	397.1 (109.0)	413.4 (116.2)	366.2 (82.0)	397.4 (115.7)	0.015	417.2 (109.5)	367.1 (101.5)	360.3 (99.8)	<0.001
Calcium, mg/MJ	85.5 (29.1)	103.3 (71.3)	79.9 (25.6)	84.1 (28.8)	0.017	89.8 (29.2)	78.6 (24.8)	78.7 (33.5)	0.002
Magnesium, mg/MJ	37.7 (8.6)	41.3 (19.0)	35.7 (7.1)	38.0 (8.7)	0.029	39.2 (8.7)	35.3 (7.6)	36.7 (8.0)	0.001
Phosphorus, mg/MJ	150.8 (32.4)	154.4 (34.6)	144.7 (29.2)	149.3 (29.3)	0.085	155.8 (33.9)	142.2 (26.0)	143.8 (32.4)	0.008
Iron, mg/MJ	1.16 (0.30)	1.19 (0.31)	1.09 (0.23)	1.18 (0.31)	0.056	1.21 (0.30)	1.08 (0.26)	1.10 (0.29)	0.003
Zinc, mg/MJ	1.12 (0.18)	1.13 (0.18)	1.10 (0.17)	1.10 (0.18)	0.271	1.15 (0.18)	1.08 (0.16)	1.08 (0.17)	0.007
Copper, mg/MJ	0.16 (0.03)	0.16 (0.03)	0.16 (0.03)	0.16 (0.03)	0.167	0.16 (0.03)	0.16 (0.03)	0.16 (0.03)	0.068
Manganese, mg/MJ	0.44 (0.14)	0.45 (0.16)	0.42 (0.13)	0.44 (0.13)	0.358	0.46 (0.16)	0.41 (0.11)	0.43 (0.14)	0.084
Retinol, μg/MJ	54.7 (31.8)	55.1 (33.0)	51.1 (30.6)	59.3 (29.9)	0.184	54.7 (33.4)	51.9 (27.3)	60.3 (32.8)	0.500
β-Carotene, μg/MJ	649.1 (469.1)	706.0 (544.3)	545.8 (304.5)	642.9 (415.6)	0.042	712.5 (526.5)	561.0 (341.1)	520.0 (329.9)	0.007
Retinol equivalent, μg/MJ	109.2 (49.9)	114.3 (54.9)	97.0 (41.7)	113.3 (43.4)	0.030	114.4 (53.4)	99.0 (40.9)	104.0 (46.7)	0.110
Vitamin D, μg/MJ	1.94 (1.31)	2.12 (1.41)	1.72 (1.00)	1.79 (1.40)	0.044	2.07 (1.40)	1.77 (0.95)	1.69 (1.43)	0.089

α-Tocopherol, mg/MJ	1.09 (0.29)	1.13 (0.31)	1.03 (0.24)	1.10 (0.29)	0.056	1.13 (0.30)	1.04 (0.26)	0.99 (0.28)	0.006
Vitamin K, µg/MJ	52.9 (27.1)	55.1 (29.4)	48.2 (20.5)	54.1 (28.7)	0.394	56.3 (28.5)	46.5 (22.5)	49.8 (26.7)	0.025
Vitamin B_1, mg/MJ	0.11 (0.02)	0.12 (0.03)	0.11 (0.02)	0.11 (0.02)	0.068	0.12 (0.02)	0.11 (0.02)	0.10 (0.02)	<0.001
Vitamin B_2, mg/MJ	0.20 (0.05)	0.20 (0.05)	0.19 (0.04)	0.20 (0.05)	0.088	0.20 (0.05)	0.18 (0.04)	0.19 (0.06)	0.005
Niacin, mgNE/MJ	2.43 (0.63)	2.49 (0.63)	2.29 (0.60)	2.47 (0.66)	0.060	2.52 (0.62)	2.25 (0.53)	2.35 (0.78)	0.009
Vitamin B_6, µg/MJ	185.3 (47.6)	192.0 (50.9)	169.6 (41.5)	182.9 (50.3)	0.005	193.7 (48.3)	167.3 (45.1)	171.3 (49.9)	<0.001
Vitamin B_{12}, µg/MJ	1.29 (0.64)	1.38 (0.67)	1.16 (0.55)	1.23 (0.69)	0.029	1.35 (0.68)	1.20 (0.48)	1.18 (0.74)	0.095
Folic acid, µg/MJ	53.2 (20.1)	55.9 (21.8)	47.8 (14.9)	53.8 (20.8)	0.016	56.8 (21.0)	47.3 (17.3)	47.7 (16.9)	<0.001
Pantothenic acid, mg/MJ	0.94 (0.19)	0.96 (0.19)	0.91 (0.18)	0.95 (0.20)	0.077	0.97 (0.19)	0.88 (0.17)	0.93 (0.22)	0.002
Vitamin C, mg/MJ	18.9 (8.3)	20.0 (8.8)	16.8 (6.5)	19.0 (8.6)	0.020	20.3 (8.5)	17.2 (8.0)	15.4 (5.9)	<0.001
Daidzein, mg/MJ	2.22 (1.31)	2.18 (1.30)	2.15 (1.25)	2.45 (1.43)	0.497	2.24 (1.32)	2.01 (1.20)	2.51 (1.45)	0.188
Genistein, mg/MJ	3.76 (2.20)	3.69 (2.19)	3.65 (2.10)	4.15 (2.41)	0.486	3.80 (2.21)	3.41 (2.02)	4.25 (2.44)	0.191
Alcohol, %E	2.7 (6.1)	2.5 (6.2)	2.2 (5.4)	2.0 (4.2)	0.985	2.7 (6.4)	1.4 (3.4)	2.4 (5.7)	0.453
